# Thrombosis and COVID-19: The Potential Role of Nutrition

**DOI:** 10.3389/fnut.2020.583080

**Published:** 2020-09-25

**Authors:** Alexandros Tsoupras, Ronan Lordan, Ioannis Zabetakis

**Affiliations:** ^1^Department of Biological Sciences, University of Limerick, Limerick, Ireland; ^2^Bernal Institute, University of Limerick, Limerick, Ireland; ^3^Health Research Institute, University of Limerick, Limerick, Ireland; ^4^Institute for Translational Medicine and Therapeutics, Perelman School of Medicine, University of Pennsylvania, Philadelphia, PA, United States

**Keywords:** nutrition, SARS-CoV-2, COVID-19, thrombosis, antithrombotic

## Abstract

Severe acute respiratory syndrome coronavirus 2 (SARS-CoV-2), responsible for the coronavirus disease (COVID-19), is a contagion that has rapidly spread around the globe. COVID-19 has caused significant loss of life and disrupted global society at a level never before encountered. While the disease was predominantly characterized by respiratory symptoms initially, it became clear that other systems including the cardiovascular and neurological systems were also involved. Several thrombotic complications were reported including venous thrombosis, vasculitis, cardiomyopathy, and stroke. Thrombosis and inflammation are implicated in various non-communicable diseases (NCDs). This is of significant concern as people with pre-existing conditions such as cardiovascular disorders, renal disorders, obesity, metabolic syndrome, and diabetes are at greater risk of severe COVID-19 infection. Consequently, the research surrounding the use of anticoagulants, antiplatelet, and antithrombotic strategies for prophylaxis and treatment of COVID-19 is of critical importance. The adoption of a healthy diet, physical exercise, and lifestyle choices can reduce the risk factors associated with NCDs and the thrombo-inflammatory complications. In this review, these thrombotic complications and potential foods, nutraceuticals, and the antithrombotic constituents within that may prevent the onset of severe thrombotic complications as a result of infection are discussed. While nutrition is not a panacea to tackle COVID-19, it is apparent that a patient's nutritional status may affect patient outcomes. Further intensive research is warranted to reduce to incidence of thrombotic complications.

## Introduction

Since December 2019, the coronavirus disease (COVID-19) has rapidly spread worldwide becoming a pandemic as declared by the World Health Organization (WHO). Over 20 million cases of COVID-19 have been reported, which is responsible for 738,000 deaths globally as of August 11th (1). COVID-19 is caused by the severe acute respiratory syndrome coronavirus 2 (SARS-CoV-2), which is an enveloped positive-sense single-stranded RNA virus infecting cells of the respiratory system *via* the angiotensin-converting enzyme 2 (ACE2) receptor, when the spike protein is primed by transmembrane protease serine 2 protein (TMPRSS2) (2). SARS-CoV-2 entry into host cells is followed by a subsequent response of the immune system, which includes inflammation-related manifestations leading to disproportionate endothelial damage and dysfunction, dysregulation of perfusion, and a loss of hypoxic vasoconstriction (3). The lungs seem to be the initial target organ of SARS-CoV-2, whereby patients develop acute lung injury that can progress to respiratory failure. Symptoms present as upper and lower respiratory tract infections that in severe cases is accompanied with inflammatory complications that are attributed to a cytokine storm and hyperinflammation (4).

The cytokine storm can result in acute respiratory distress syndrome (ARDS) and potentially multiorgan failure (5, 6). Infection of the respiratory epithelium is the portal of entry, whereby alveolar damage may be mediated by endothelial injury, resulting in the release of cytokines and chemokines, the recruitment of immune cells, and the activation of coagulation and thrombosis (6–11). The intense cytokine storm is also implicated in the virus-triggered pulmonary tissue damage, functional impairment, reduced lung capacity, and respiratory failure in severe COVID-19 cases. SARS-CoV-2 may extend to other organs as documented by reports that patients with severe COVID-19 often develop neurological issues and multiorgan damage including cardiac injury and acute kidney injury (6, 7, 12). Indeed, it is now known that other complications such as hypercoagulability, venous thrombosis, systemic vasculitis, and stroke are associated with increased mortality in COVID-19 patients (12, 13). Therefore, identifying, repurposing or designing therapeutics and preventative strategies to reduce the risk of thrombotic complications in COVID-19 is of significant importance.

Currently, there are no known effective prophylactic treatments for COVID-19 and only limited effectiveness reported for various therapeutics such as Remdesivir, which reduces the time to recovery in hospitalized COVID-19 patients (14). Press releases and preprints occasionally have shed light on effective treatments such as dexamethasone (15), but many other potential therapeutics that have come to light have yet to be peer reviewed and require much further study. Likewise, the effectiveness of antithrombotic and anticoagulant treatments is still under investigation. In this mini review, the evidence surrounding thrombosis and how nutrition may play a role in the primordial prevention of the development of thrombotic complications in SARS-CoV-2 infected patients is discussed. Several nutrients that exhibit antiplatelet activity are summarized and future directions of nutrition research as a strategy to reduce thrombotic complication and severe infection of COVID-19 are suggested.

## SARS-CoV-2 and Thrombotic Complications

Non-communicable diseases (NCDs) seem to be associated with higher incidences of severe COVID-19 infections (7, 16, 17). This is due to the presence of comorbidities and risk factors amongst these subpopulations who may be obese, elderly, or immunocompromised (7, 18). Many NCDs are associated with thrombotic complications such as cardiovascular diseases (CVD), acute coronary syndromes, ischemic stroke, deep vein thrombosis, venous thromboembolism, and pulmonary embolism (19, 20). While thrombosis is a complication of COVID-19 (13), the presence of NCDs such as diabetes or obesity may further increase the risk of thrombotic complications in severely ill patients due to their associations with a prothrombotic state (21). Generally, the hemostatic system that is comprised of coagulation, platelet aggregation, and fibrinolysis, is a host defense mechanism that maintains the integrity of the circulatory system following vascular damage (19, 22). The coagulatory and inflammatory processes are highly regulated and are crucial to the host defense systems for limiting tissue injury, removing pathogens, and re-establishing homeostasis (10, 22).

Pulmonary embolism and thrombosis are now known to contribute to adverse events and increased mortality in critically ill COVID-19 patients due hypercoagulability and a prothrombotic state that can lead to coagulopathy and disseminated intravascular coagulation (11). Therefore, widespread pulmonary and endothelial inflammation and enhanced thrombosis are integral features of COVID-19 pathology. This state of hypercoagulation contributes to a fatal deterioration of the ventilation/perfusion ratio (V/Q), which is a measure of the concentration of air reaching the alveoli per minute to the amount of blood reaching the alveoli per minute *via* the capillaries (8). This is of clinical importance as hypoxia and sepsis can independently promote platelet aggregation mediated by the release of von Willebrand factor, which is increased in the whole blood of intensive care unit (ICU) patients with COVID-19 (8, 23). However, further study is required as only a limited number of patients were enrolled in this study. The substantial coagulation activation in severe COVID-19 infection is likely related to the sustained inflammatory response due to the intense cytokine release induced by virus invasion. Increased levels of D-dimers, a prolongation of prothrombin time (PT), activated partial thromboplastin time (aPTT), and thrombocytopenia is representative of disease severity and have been correlated with poor prognosis and increased mortality in COVID-19 patients (11, 24). However, whether the hemostatic changes observed are specific to COVID-19 or are a reflection of the cytokine storm and systemic inflammation is still subject to debate. Nevertheless, these changes in hemostatic factors seems to resemble that of inflammatory markers including IL-6, which potentially indicates a severe inflammatory response accompanied by a secondary hypercoagulable state (10, 24). Indeed, the loss of normal antiplatelet and anti-inflammatory functions of endothelial cells also leads to dysregulation of platelet activation, coagulation, and leukocyte recruitment in the microvasculature, with complement activation possibly playing an important role in the context of COVID-19-associated pneumonitis and purpuric skin lesions (6, 24).

Proteomic and metabolomic characterization of COVID-19 patient sera compared to control groups revealed molecular changes in COVID-19 patients demonstrating dysregulation of macrophages, evidence of platelet degranulation, the involvement of complement system pathways, and significant metabolic suppression, which may be useful as potential blood biomarkers for disease severity evaluation (25). Vascular inflammation and viral endotheliitis are frequent in severe COVID-19 patients as a result of endothelial dysfunction. Large increases of von Willebrand factor (VWF) and factor VIII activity has also been observed in COVID-19, which is also attributed to endothelial damage (26, 27). Notably, even in the absence of severe disease, patients with COVID-19 may be at heightened risk of thrombus formation leading to stroke due to viral interaction with the endothelium (28). It was reported in New York City that some patients experiencing mild symptoms presented with stroke and large vessel thrombosis without occlusion. Whereas, severely infected patients presented with venous thrombosis and microangiopathy (28, 29).

In general, viral infections can activate monocytes, tissue macrophages, and endothelial cells, thus triggering the production of proinflammatory cytokines and activation of the coagulation cascade. Inflammation and coagulation synchronously respond to invading pathogens as part of the host's defense system, which includes complex processes referred to as thrombo-inflammation or immunothrombosis. In this capacity platelets can become activated following antigen recognition. Activated platelets can directly gather and inactivate pathogens and/or facilitate the clearance of pathogenic microorganisms by promoting the formation of neutrophil extracellular traps (NETs), activating neutrophils and macrophages, and facilitating the formation of platelet aggregates and microthrombi (30), all of which contribute to the crosstalk between the inflammation and coagulation pathways (7).

The complement system is part of the innate immune system and represents one of the first responses of the host immune system to SARS-CoV-2 infection *via* activation of multiple pathways (31). As a result of complement activation, there is significant platelet activation, thrombus formation, endothelial dysfunction, and intravascular coagulation, that can culminate in multiorgan failure and death in severe COVID-19 infection (6). It is hypothesized that C5a and C5b-9 that are generated by cells infected with SARS-CoV-2 may be key mediators of COVID-19-associated endothelial dysfunction and platelet activation. Activation of these cells may lead to the exocytosis of P-selectin and von Willebrand factor multimers and the expression of tissue factor and adhesion molecules from endothelial cells, and the release of chemokines and platelet-activating factor (PAF), which together promote inflammation, increase vascular permeability, and trigger the coagulation process (6, 32). C5b−9 can also be a powerful platelet agonist by inducing the secretion of storage granules and the release of procoagulant platelet microparticles leading to vascular injury and dysfunction followed by the formation of significant blood clots (6, 32).

Overall, COVID-19 patients have a higher risk for thromboembolic complications and a higher frequency is observed in severely ill patients, which can lead to significant damage (33). As a result, thrombotic complications are a potential target for the reduction of disease severity.

## Mitigation of COVID-19 Thrombotic Complications

To avoid thrombotic complications, many have recommended prophylactic antithrombotic pharmacological therapies for COVID-19 patients, along with particular dosing instructions (34–37). However, to date, little research has been conducted and the optimal doses of anticoagulants or antiplatelet agents has yet to be determined. Indeed, data for prospective efficacy and safety of existing antiplatelet therapies to treat or prevent severe symptoms of COVID-19 remains elusive. Despite that, some institutes have adopted pharmacological thromboprophylactic strategies (38). These include the use of intermediate or full-dose preemptive anticoagulation therapy rather than prophylactic dosing for routine care of COVID-19 patients (13). Considering some studies have observed high bleeding rates as a result of standard anticoagulation treatment in COVID-19 patients, randomized trials are required to determine any potential benefit of intensified anticoagulant prophylaxis in COVID-19 patients (39).

One potential therapeutic avenue has shown that antiplatelet treatments can improve hypoxemia in severe COVID-19 patients with hypercoagulability by affecting the ventilation/perfusion ratio in patients with severe respiratory failure (8). However, antiplatelet and anticoagulant therapies administered pre-admission to hospital does not seem to protect with ARDS at presentation who are at significant risk of death (35). Pharmacological agents targeting thrombo-inflammation in COVID-19, including antiplatelet compounds, along with proposed best practices and clinical guidance, has been extensively reviewed elsewhere (34, 36, 37). In brief, current guidelines proposed by the International Society of Thrombosis and Hemostasis recommend low-molecular weight heparin for both mild and severely infected patients who required hospitalization (40). However, further guidance for the utilization of antithrombotic and antiplatelet therapies in patients with known or suspected COVID-19 are necessary, particularly for patients with comorbidities (36).

## Foods and Nutrients With Antithrombotic Properties

Diet and lifestyle are modifiable risk factors that can have a significant impact on an individual's likelihood to develop an NCD (41, 42) and their susceptibility to developing infections (7). It has previously been described how inflammation and thrombosis are implicated in the onset and progression of NCDs (19, 41). However, it has become apparent that one's nutritional status is an important factor for priming the immune system to tackle acute infections such as COVID-19 (7, 43–45). Adoption of healthy dietary habits will prevent the onset of NCDs, which is a significant risk factor for the development of COVID-19, and may provide support to the immune system to lessen the severity of an infection (7). A healthy diet in compliance with current nutritional recommendations can lead to less societal and economic burden to health systems and economies (7, 46). Therefore, promotion of a healthy diet and lifestyle among the general population characterized by anti-inflammatory and antithrombotic properties may potentially benefit or prevent the thrombo-inflammatory manifestations of patients with NCDs and comorbidities that can significantly impact the outcomes of COVID-19 patients (7, 36, 47).

Healthy dietary patterns such as the Mediterranean diet or the DASH diet (Dietary approaches to reduce hypertension) are characterized by high intakes of fruit and vegetables, whole grains, fermented foods, moderate intake of fish, dairy, and low intake of processed foods (48, 49). Indeed, health outcomes in relation to adherence to the Mediterranean diet during the COVID-19 pandemic is currently under investigation (ClinicalTrials.gov: NCT04449731). Notably, most national dietary guidelines support these same nutritional patterns, whereby high fruit, vegetable, nuts, dairy, and fish intake is promoted (48). These foods are rich in bioactive compounds with potential antithrombotic and anti-inflammatory properties as highlighted in Table 1 (41, 60, 82–85). These include phytochemicals such as phenolic compounds, carotenes, alkaloids, terpenes, peptides, and bioactive lipid molecules. The latter group of molecules includes monounsaturated fatty acids (MUFA; e.g., oleic acid), omega-3 polyunsaturated fatty acids (e.g., n-3 PUFA; alpha linolenic acid, eicosapentaenoic acid, and docosahexaenoic acid), lipid soluble vitamins (vitamin D and vitamin E), and bioactive polar lipids (e.g., phospholipids, sphingolipids, and glycolipids) (48). Many of these compounds exhibit potent inhibition or modulation of several signaling pathways of key pro-inflammatory and prothrombotic mediators such as platelet-activating factor (PAF), thrombin, collagen, ADP, arachidonic acid, and related eicosanoids (41, 50, 60).

**Table 1:** A selection of some of the main food-derived bioactive constituents that can affect platelet activity that may be of value to prevent thrombotic complications in COVID-19.

**Antithrombotic constituents**	**Main dietary sources**	**Some of the documented antiplatelet effects**	**Further reading**
**Bioactive lipids compounds**			
- Polar Lipids	- Fish and seafood - Dairy - Olives and olive oil - Tea - Meat - Wine and fermented beverages	- Reduction of PAF -induced platelet aggregation *in vitro, in vivo*, and *ex vivo* - Potential anti-inflammatory effects - Affects PAF metabolism *in vitro, in vivo*, and *ex vivo* - Reduction of thrombin, ADP, and collagen -induced platelet aggregation *in vitro*	(41, 50–59)
- n-3 PUFA	- Fish and seafood - Nuts and seeds - Plant oils	- Reduced thrombin formation - Reduced oxidative stress - Reduced Lp-PLA_2_ - Anti-inflammatory - Immunomodulatory	(60–65)
- Vitamin E	- Plant-derived oils (e.g., olive oil), nuts, and seeds	- Reduction of PAF, ADP, thrombin, and collagen-induced platelet aggregation in PRP and whole blood *in vitro* and *ex vivo* - Reduction of PAF and prostaglandin synthesis	(66–69)
- Vitamin D	- Dairy - Oily fish - Meat	- Reduction of PAF platelet aggregation and metabolism *in vitro* and *in vivo*- Anti-inflammatory - Low vitamin D associated with higher platelet reactivity in diabetics	(70, 71)
**Bioactive Peptides**	- Fish and seafood - Dairy - Seaweeds - Cereal grains	- Inhibition of COX-1 - Inhibition of type-III collagen-induced aggregation - Binding to glycoprotein IIb/IIIa - Interactions with fibrinogen and its receptor. - Reduction of thrombin formation - Reduction of ADP-induced platelet aggregation - Anti-inflammatory	(72–75)
**Phytochemicals, vitamins, and phenolic compounds**			
- Phytosterols - Phytostanols - Flavonoids - Isoflavonoids - Anthocyanins - Phenolipids - Alkaloids	- Fruit and berries - Vegetables - Seeds, nuts, and oils - Plant oils - Wine - Tea - Fermented beverages	- Reduction of platelet aggregation - Anti-inflammatory effects - Affects PAF metabolism *in vitro, in vivo*, and *ex vivo* - Reduction of tissue factor synthesis - Regulation of endothelial function - Inhibition of COX and LOX pathways	(41, 54, 76–81)

*ADP, adenosine diphosphate, COX-1, cyclooxygenase-1; Lp-PLA_2_, lipoprotein-associated phospholipase A_2_; PAF, platelet-activating factor; PRP, platelet-rich plasma*.

Dietary polar lipids found in a variety of foods (Table 1) are of particular interest as they exhibit potent *in vitro* and *in vivo* anti-PAF effects, which can reduce PAF-induced activation platelets and other cells including leukocytes and endothelial cells. Indeed, there is also evidence that polar lipids can reduce the activities of PAF anabolic enzymes and in increase the levels of PAF catabolic enzymes, which leads to a reduction in the overall levels of PAF. Along with modulating PAF metabolism, these molecules may also reduce the oxidation of plasma lipoproteins, thus avoiding a PAF-related cascade of synthesis and expression of other chemokines, cytokines, and their respective receptors and pathways, which are involved in thrombosis, coagulation, and thrombo-inflammation (41, 51, 86, 87). For instance, nutritional trials involving the Mediterranean diet have been shown to reduce the risk for NCDs by reducing PAF activity in blood (88, 89). This is particularly interesting as early metabolomics data indicates that there is a significant shift in the glycerophospholipid composition of patient sera, which are related to platelet degranulation and may be related to PAF-like lipids (25). Other lipids such as n-3 PUFA are also known to exhibit antithrombotic, anti-inflammatory, and pro-resolving effects that may be beneficial against SARS-CoV-2 (7, 90). However, their bioavailability and efficacy is subject to debate (61). Further clinical trials are required to determine if n-3 PUFA and anti-PAF compounds have a functional consequence against thrombotic complication in acute infections.

Food-derived bioactive peptides are also functionally important as they exhibit antihypertensive, antioxidant, antithrombotic, anti-inflammatory properties, and immunomodulatory properties (72). Bioactive peptides found in protein hydrolysates generally contain 2–20 amino acid residues (91). They have a low molecular weight, high bioavailability, and their molecular structures allows them to readily interact with different proteins and their receptors (72). Bioactive peptides from foods such as dairy products, seafood, seaweeds, nuts, cereals, and other natural sources possess antithrombotic properties that may be useful as antiplatelet agents as they seem to interact with multiple platelet activation pathways. These include inhibition of cyclooxygenase-1 (COX-1), inhibition of type-III collagen-induced aggregation, binding to glycoprotein IIb/IIIa, interacting with fibrinogen and its receptor, and affecting thrombin (73, 74).

Other food constituents such as phenolic compounds found in fruit, vegetables, cereals, and fermented beverages such as wine and beer also beneficially affect platelet function as outlined in Table 1. Indeed, resveratrol is thought to be one of the main bioactive components of wine responsible for its antiplatelet effects (92, 93), which is currently under investigation as a prophylactic and therapeutic in COVID-19 patients (ClinicalTrials.gov: NCT04400890). Herbal phenolic compounds in conjunction with colchicine is also being investigated for potential anti-inflammatory effects in COVID-19 patients (ClinicalTrials.gov: NCT04392141). However, while many of the molecules described in this review are under investigation for their prophylactic and therapeutic properties against SARS-CoV-2, none of the outcomes measured relate to thrombosis yet.

Generally, only a limited number of food-derived compounds have been investigated for their antiplatelet effects clinically as of yet, whereby fish oils are synonymous. However, there has been some success in developing antiplatelet nutraceuticals. One such product is a water-soluble tomato-based nutraceutical given the trademarked name Fruitflow®. Fruitflow is now recognized as a functional product by the European Food Safety Authority (EFSA) with a recognized health claim that states that Fruitflow® “helps maintain normal platelet aggregation, which contributes to healthy blood flow” (94). A 3 g dose (containing 65 mg of antiplatelet components) of Fruitflow® recently demonstrated antiplatelet effects that were approximately one-third the effect of 75 mg of aspirin in a double-blinded randomized clinical trial of 47 healthy subjects (95). Nutraceuticals such as these may serve as a safe antiplatelet prophylactic treatment for those at high risk of COVID-19 who may also be at increased risk of thrombotic complications and an alternative to pharmacological compounds that may cause greater risk of bleeding (19).

In Table 1, there are several bioactive lipids, peptides, phytochemicals, and phenolic compounds that can affect platelet activity that may have the capacity to prevent the negative thrombotic outcomes of COVID-19. While these constituents are not the panacea to treat COVID-19, the adoption of a healthy diet characterized by many of these molecules along with a healthy lifestyle may help maintain hemostasis and prevent thrombotic complications. While research is on-going to find an effective pharmacological treatment for COVID-19, it is important to consider that the nutritional status of a patient may affect their health outcomes. Indeed, future COVID-19 studies must consider the effect of nutrition on factors relating to thrombosis and inflammation and how might nutrition play a role in the mitigation of potential vascular and thrombotic complications. Nutritional strategies and the use of nutraceuticals must be further investigated for their potential to prevent thrombotic complications in COVID-19 patients. Clinical trialists currently investigating some of the compounds mentioned in Table 1 in conjunction with various therapies for other COVID-19 targets should consider the inclusion of measurable thrombosis-related parameters within their existing or planned trials. While there is no evidence that consuming nutritional supplements might protect anyone from becoming infected with COVID-19 or preventing its thrombotic complications, it is imperative that national health authorities promote a healthy diet and lifestyle to maintains one's nutritional status and wellbeing as the pandemic evolves.

## Conclusions

It is evident that thrombotic complications are a significant risk factor for COVID-19 patients. Therefore, there is an urgent need to establish further clinical trials to investigate potential pharmacological and nutritional mitigation strategies to prevent thrombotic complications as a result of severe COVID-19 infection. It is also important to discern whether prophylactic antiplatelet therapies are appropriate in the case of initially mild infections or those with comorbidities and NCDs to prevent potential thrombosis leading to stroke or other major complications. Indeed, it is important that clinical trialists currently investigating some of the compounds mentioned in Table 1 for other COVID-19 targets include measurable thrombosis-related parameters within their existing or planned trials. While, some institutes have endorsed guidelines for antithrombotic and antiplatelet strategies, these have yet to be verified as effective by clinical study. For those non-infected and especially for those with or at increased risk of underlying NCDs, the adoption of a healthy diet and lifestyle may prevent the onset of severe thrombotic complications due to the presence of bioactive compounds with antiplatelet effects. Coupling a healthy diet and lifestyle along with preventing infection by following social distance guidelines, wearing a mask, and adopting good public hygiene with prevent the onset of severe infection.

## Author Contributions

All authors listed have made a substantial, direct and intellectual contribution to the work, and approved it for publication.

## Conflict of Interest

The authors declare that the research was conducted in the absence of any commercial or financial relationships that could be construed as a potential conflict of interest.
